# Determinants of Sugar-Sweetened Beverage Consumption Among the Saudi Adults: Findings From a Nationally Representative Survey

**DOI:** 10.3389/fnut.2022.744116

**Published:** 2022-03-22

**Authors:** Mohammed Khaled Al-Hanawi, Moin Uddin Ahmed, Noor Alshareef, Ameerah Mohammad Nour Qattan, Mohammad Habibullah Pulok

**Affiliations:** ^1^Department of Health Services and Hospital Administration, Faculty of Economics and Administration, King Abdulaziz University, Jeddah, Saudi Arabia; ^2^Health Economics Research Group, King Abdulaziz University, Jeddah, Saudi Arabia; ^3^Translational Health Research Institute, Western Sydney University, Campbelltown, NSW, Australia; ^4^Institute of Health Economics, University of Dhaka, Dhaka, Bangladesh; ^5^Geriatric Medicine Research, Nova Scotia Health Authority, Halifax, NS, Canada; ^6^School of Health Administration, Dalhousie University, Halifax, NS, Canada

**Keywords:** adults, Saudi Arabia (KSA), sugar-sweetened beverage, calories, fast food

## Abstract

**Background:**

Saudi Arabia is the fifth largest consumer of calories from sugar-sweetened beverages (SSBs) in the world. However, there is a knowledge gap to understand factors that could potentially impact SSB consumption in Saudi Arabia. This study is aimed to examine the determinants of SSBs in Saudi Arabia.

**Methods:**

The participants of this study were from the Saudi Health Interview Survey (SHIS) of 2013, recruited from all regions of Saudi Arabia. Data of a total of 10,118 survey respondents were utilized in this study who were aged 15 years and older. Our study used two binary outcome variables: weekly SSB consumption (no vs. any amount) and daily SSB consumption (non-daily vs. daily). After adjusting for survey weights, multivariate logistic regression models were applied to assess the association of SSB consumption and study variables.

**Results:**

About 71% of the respondents consumed SSB at least one time weekly. The higher likelihood of SSB consumption was reported among men, young age group (25–34 years), people with lower income (<3,000 SR), current smokers, frequent fast-food consumers, and individuals watching television for longer hours (≥4 h). Daily vegetable intake reduced the likelihood of SSB consumption by more than one-third.

**Conclusions:**

Three out of four individuals aged 15 years and over in Saudi Arabia consume SSB at least one time weekly. A better understanding of the relationship between SSB consumption and demographic, socioeconomic, and behavioral factors is necessary for the reduction of SSB consumption. The findings of this study have established essential population-based evidence to inform public health efforts to adopt effective strategies to reduce the consumption of SSB in Saudi Arabia. Interventions directed toward education on the adverse health effect associated with SSB intake are needed.

## Introduction

Sugar-sweetened beverages (SSBs) are the most widely consumed caloric beverage (1–3). They contain a high amount of added sugar, calorie, and energy and are poor in nutritional value (3–5). According to the World Health Organization (WHO), SSB is defined as “all types of beverages containing free sugars, and these include carbonated or non-carbonated soft drinks, fruit/vegetable juices and drinks, liquid and powder concentrates, flavored water, energy, and sports drinks, ready-to-drink tea, ready-to-drink coffee, and flavored milk drinks” (6). Reducing SSB consumption is recommended by the WHO (7) and documented in the 2015–2020 dietary guidelines for people of the United States of America (USA) (8). SSBs are the largest source of added sugar and a key source of energy intake among both children and adults in the USA (9). Globally, excess sugar consumption has become an important public health concern (10).

SSB consumption levels among adults vary widely across countries. For instance, in the USA, a decreased trend in consumption of SSB was found between 1999 and 2010 in both youth and adults (11). In Australia, however, in 2011–12, national data indicated that 50% of Australians drank SSB on the day before the interview (12). SSB intake is particularly concerning among Saudi adults. According to Banajiba and Mahboub, Saudi Arabia is considered one of the biggest consumers of sugary drinks in the middle-eastern region (13). The country also has high prevalence rates of overweight, obesity, and diabetes. A growing amount of evidence from experimental trials and observational studies showed that regular SSB intake resulted in excess weight gain and might contribute to obesity and type 2 diabetes (9). Evidence also indicated that reducing the consumption of SSB will have a substantial impact on the prevalence of obesity and obesity-related diseases, such as diabetes (9).

A number of studies explored determinants of high SSB consumption among adults. Those studies reported that gender, age, socioeconomic status, marital status, and educational level were positively associated with SSB consumption (14–17). The majority of prior research also showed that an unhealthy lifestyle (e.g., poor-quality diet, sedentary behaviors, and smoking) was associated with higher SSB consumption intake. A poor-quality diet, specifically, lower intake of fruits and vegetables, infrequent breakfast consumption, high intake of meat, fast and fried food consumption (16, 18–20), and sedentary behaviors, such as prolonged television watching and lack of physical activity, were linked to more SSB intake (14, 16, 21). Other determinants, such as inadequate household food supply, was also associated with higher SSB consumption (22).

Many studies assessing the association between SSB intake and overweight, obesity, type 2 diabetes mellitus, cardiovascular diseases, and depressive symptoms found a positive association, while other studies found none (2, 23–26). Thus, it is imperative to understand the determinants influencing the consumption of SSB to inform future intervention development. Therefore, this study aimed to assess the determinants of SSB in the Kingdom of Saudi Arabia (KSA) using national survey data. This study has two objectives: (1) to determine the prevalence of SSB consumption and (2) to examine the association between SSB consumption and an array of demographic, socioeconomic, and behavioral factors. By understanding the determinants of SSB consumption, strategies aimed at curbing intake can be crafted to guide decisions effectively. This is of particular importance when designing strategies geared toward high-risk groups. The findings of this study can also help raise awareness about health problems that are linked to excessive consumption of added sugars in beverages, which might prompt behavioral intention toward reducing sugary drinks intake.

## Materials and Methods

### Data Source

This study obtained secondary data from the 2013 Saudi Health Interview Survey (SHIS) for empirical analysis. The SHIS was representative of the Saudi population aged 15 years and older in 2013 (27). The Saudi Ministry of Health (SMOH) implemented this survey across all the regions of Saudi Arabia between April and June 2013. The Institute for Health Metrics and Evaluation (IHME) at the University of Washington provided technical support to the SMOH for conducting the SHIS. The Government of the KSA provided financial assistance to complete the data collection. This survey was designed following the USA's National Health and Nutrition Examination Survey (NHANES). The goal of the SHIS was to collect information about socioeconomic, demographic, health characteristics, the use of health services, and health-related behavior of the Saudi population. Trained interviewers collected data using a structured questionnaire in a face-to-face interview, and DatStat software was used in data collection (28). The reliability of data collection was ensured by giving real-time feedback to the interviewers and supervisors in the field.

### Survey Design

The SHIS was a cross-sectional household survey, where participants were selected through a multistage stratified random sample technique for ensuring the representativeness of the Saudi population. Thirteen administrative regions of the KSA formulated the strata of this survey. Primary sampling units (PSUs) of the survey were selected based on enumeration household units or clusters (on average, each cluster has about 140 households) from the Census Bureau of Saudi Arabia (28). PSUs were randomly selected according to the probability proportional to size sampling approach. This process randomly selected 12,000 households from all these PSUs (14 households in each PSU). Further descriptions of the sampling methods and data collection procedures are available in prior publications (27, 29). Sample weights were integrated with the datasets for statistical analyses.

### Study Sample

The response rate was about 90% in this survey (28), which led to interviewing 10,735 respondents aged ≥15 years. We limited our study to only participants with no missing data on SSB consumption. About 3.3% of the observations had no data on SSB consumption. We discarded observations with missing data on SSB consumption. This restriction gave us an analytic sample of 10,118 individuals. Out of 10,118 individuals, 5,028 (49.7%) participants were men and 5,090 (51.3%) participants were women.

### Outcome Variables

To measure the consumption of SSB, respondents in the SHIS were asked the following questions: (1) In a typical week, how many days do you drink regular soda or pop that contains sugar, sweetened iced teas, sports drinks, or fruit drinks? (Do not include diet soda, sugar-free drinks, or 100% pure fruit juice) and (2) How many servings of SSBs did you drink on one of those days? A binary outcome variable was constructed based on these questions, which equals 1 if the study participants consume any SSB in a typical week and zero otherwise. Following the previous literature (30), a second outcome variable was constructed as regular/daily consumers of SSB reported consuming at least one SSB every day in the past week prior to the survey.

### Explanatory Variables

Previous studies in different contexts found a wide range of variables that could be correlated with the consumption of SSB (17, 31, 32). This study followed previous literature to select the predictors of SSB consumption (17, 31, 32). These included i) demographic factors, such as sex and age, ii) socioeconomic factors, such as household income and education, iii) behavioral risk factors, such as physical activity, fast food consumption, smoking status, and iv) clinical factors, such as body mass index (BMI), diabetes, and co-morbidities. Table 1 provides details on the description and construction of the variables. Age was categorized into seven groups following the SHIS (33). Education was classified into three groups. Daily fruit and vegetable intake was categorized into three groups: 0–2 serves, 3–4 serves, and 5 or more serves (22). Smoking status was categorized into three groups: never smoker, ex-smoker, and current smoker (17). Physical activity was assessed by asking the respondents about the time he or she spent doing different types of activities in a typical week. These activities included vigorous-intensity activity (large increases in breathing or heart rate), moderate-intensity activity (small increases in breathing or heart rate), walking to work and other places, and recreational activities (e.g., sports and fitness training). Physical activity was categorized into four groups: not active, low, moderate, and high (30, 33). A total of fewer than 150, 150–450, and more than 450 min of moderate to intense activity each week were considered low, moderate, and high levels of physical activity, respectively (33). BMI was categorized by following the WHO guideline (34).

**Table 1:** Description of independent variables.

	**Variable**	**Measurement scale**	**Description**
Demographic variables	Age	Categorical	Seven age groups were recoded as 15–24,25–34,35–44,45–54,55–64, and 65 years or above, with 15–24 years as the reference group.
	Sex	Binary	Sex was recoded into a dummy variable (female = 0 and male = 1) with female as reference group.
	Marital status	Categorical	Marital status was divided into three groups: currently married (reference), Separated/widowed/divorced and never married
	Region	Categorical	Regions were categorized into 13 regions, with Riyadh as reference category.
Socio-economic variables	Education	Categorical	Education had three groups: Primary or less (reference), intermediate/secondary/technical and tertiary.
	Household Income	Categorical	Household income (in Saudi Riyal) was classified into six categories: <3,000 (reference), 3,000–5,000, 5,000–7,000, 7,000–10,000, 10,000–15,000 and 15,000+. Income is reported in Saudi Riyal (SR) (1 US = 3.75 SR).
	Employment status	Categorical	Employment status was recoded as employed (reference), unemployed and not in the labor force.
Health behaviors variables	Smoking status	Categorical	Smoking status was grouped as never smoker (reference), ex-smoker, and current smoker.
	Fast food consumption	Categorical	Weekly fast food consumption was recorded as none (reference), 1–2 times and more than twice.
	Attitude toward fast food	Categorical	Individuals' attitude toward fast food was recoded as not healthy (reference), healthy, and do not know.
	Fruit intake	Categorical	Daily fruit intake is categorized into 0–2 serves (reference), 3–4 serves, 5 or more.
	Vegetables intake	Categorical	Daily vegetables intake was categorized into 0–2 serves (reference), 3–4 serves, 5 or more.
	Physical activity	Categorical	Physical activity was categorized into four groups: not active (reference), low, moderate, and high.
	Screen time	Categorical	Daily television watching time was coded as 0 to <2 h, 2 to <4 h, and 4 h and above.
Clinical variables	Body Mass Index (BMI)	Categorical	BMI was calculated as weight (kg)/height^2^ (m^2^). It is categorized into less than 25, 25–30, and more than 30 (inclusive).
	Diabetes	Binary	Self-reported diabetes status with 0 = no (reference) and 1 = yes

### Statistical Methods

This study used Stata version 16.1 for Windows (Stata Corp. 2017, Stata Statistical Software: Release 16. College Station, TX, USA: Stata Corp LLC) to implement all the statistical analyses. A total number of people who consumed any SSB (in a typical week) was divided by the total sample size to estimate the prevalence of SSB consumption. Descriptive analyses were undertaken to investigate the association between the prevalence of SSB consumption and respondents' characteristics. This association was measured using Pearson's chi-square test (35). We examined the same relationships through multivariate regression analysis, controlling for the effect of other variables. Logistic regression models were used in the multivariate analysis. The findings were presented using adjusted odds ratios (AORs) with 95% Confidence intervals (CIs) and *p*-values.

We also conducted a subgroup (male vs. female and 15–24 years age group vs. 25+ years age group) analysis. Data were weighted by the inverse of the person's probability of selection and the response rate in the regions of the KSA. We used the “*svy*” prefix command of the Stata software to account for the statistical design of the survey (weighting, clustering, and stratification). Multiple imputation method was implemented using “*mi”* command in Stata to impute missing data on independent variables. A significance level of *p* <0.05 was applied to measure the association in the multivariate models.

### Ethics Statement

The Institutional Review Board (IRB) of the SMOH reviewed and granted ethics approval for the SHIS (36). Informed consent was obtained from the participants to be included in the survey. Data were fully anonymized by the SMOH. The authors received de-identified data for this study after complying with the regulations of secondary analysis. There was no possibility to identify people using the current data. Therefore, separate ethics approval was not required for the current study.

## Results

Table 2 presents demographic, socioeconomic, clinical, and lifestyle risk factors and attitude and knowledge characteristics of the study participants by SSB consumption status. About 71% of respondents consumed SSB at least once in a typical week. The prevalence of daily consumption (i.e., regular consumer) of SSB was about 36% among the consumers. Overall, the study sample was almost equally distributed between men (51.1%) and women (48.9%). SSB consumption was significantly correlated with all the variables shown in Table 2. About 54% of the consumers (consume SSB at least once a week) were men.

**Table 2:** Respondent characteristics and sugar-sweetened beverage (SSB) consumption.

**Prevalence of SSB-consumer: 71.2%**
**Prevalence of daily consumer: 35.5%**	
	**Unweighted frequency**			**Weighted percentage**			
	**Non-consumer**	**Consumer**	**Total**	**Non-consumer**	**Consumer**	**Total**	***p*** **value**
**Overall**							
**Age**							<0.001
15–24 years	263	2,015	2,278	18.2%	48.4%	40.9%	
25–34 years	514	2,082	2,596	17.1%	23.0%	21.5%	
35–44 years	658	1,533	2,191	18.7%	14.0%	15.1%	
45–54 years	601	802	1,403	21.5%	9.1%	12.2%	
55–64 years	437	333	770	14.3%	3.5%	6.2%	
65+ years	506	299	805	10.2%	2.1%	4.1%	
Total	2,979	7,064	10,043	100.0%	100.0%	100.0%	
**Sex**							<0.001
Female	1,681	3,409	5,090	57.6%	46.0%	48.9%	
Male	1,331	3,697	5,028	42.4%	54.0%	51.1%	
Total	3,012	7,106	10,118	100.0%	100.0%	100.0%	
**Marital status**							<0.001
Currently married	2,244	4,323	6,567	69.3%	42.1%	48.9%	
Separated/Widowed/Divorced	423	396	819	9.1%	3.2%	4.6%	
Never married	338	2,369	2,707	21.5%	54.7%	46.5%	
Total	3,005	7,088	10,093	100.0%	100.0%	100.0%	
**Region**							<0.001
Riyadh	437	1,279	1,716	19.5%	25.1%	23.7%	
Western Region	377	1,103	1,480	18.4%	21.1%	20.4%	
Madinah	297	369	666	11.8%	5.6%	7.1%	
Qaseem	181	271	452	8.8%	6.3%	6.9%	
Eastern region	210	554	764	15.5%	17.9%	17.3%	
Asser/Bisha	388	559	947	7.9%	5.7%	6.2%	
Tabouk	132	488	620	1.9%	2.7%	2.5%	
Hail	180	454	634	3.2%	3.2%	3.2%	
Northern borders	133	375	508	1.1%	1.5%	1.4%	
Jazan	230	389	619	6.5%	4.6%	5.1%	
Najran	172	494	666	1.9%	2.2%	2.1%	
Albaha	148	438	586	1.5%	2.0%	1.9%	
AlJouf/Quriat	127	333	460	2.0%	2.1%	2.1%	
Total	3,012	7,106	10,118	100.0%	100.0%	100.0%	
**Education**							<0.001
Primary or less	1,406	1,669	3,075	41.2%	21.2%	26.2%	
Intermediate, secondary, or technical	997	3,655	4,652	38.3%	58.1%	53.2%	
Tertiary education	605	1,772	2,377	20.4%	20.7%	20.6%	
Total	3,008	7,096	10,104	100.0%	100.0%	100.0%	
**Household income**							<0.001
<3,000 SR	523	924	1,447	19.0%	16.5%	17.2%	
3,000 to less than 5,000 SR	433	1,128	1,561	18.4%	20.5%	20.0%	
5,000 to less than 7,000 SR	361	1,025	1,386	13.4%	15.4%	14.9%	
7,000 to less than 10,000 SR	451	1,114	1,565	20.2%	18.2%	18.7%	
10,000 to less than 15,000 SR	368	964	1,332	14.6%	15.5%	15.3%	
≥15,000 SR	336	723	1,059	14.4%	13.8%	14.0%	
Total	2,472	5,878	8,350	100.0%	100.0%	100.0%	
**Employment status**							<0.001
Employed	992	2,892	3,884	30.9%	32.8%	32.4%	
Unemployed	522	802	1,324	16.5%	10.0%	11.6%	
Not in the labor force	1,485	3,394	4,879	52.6%	57.2%	56.0%	
Total	2,999	7,088	10,087	100.0%	100.0%	100.0%	
**BMI**							<0.001
less than 25	776	2,529	3,305	30.1%	44.7%	41.0%	
25–30	1,024	2,205	3,229	33.3%	29.2%	30.2%	
30 or more	1,126	2,147	3,273	36.5%	26.1%	28.7%	
Total	2,926	6,881	9,807	100.0%	100.0%	100.0%	
**Smoking status**							<0.001
Never	2,579	5,792	8,371	85.6%	83.1%	83.8%	
Ex-smoker	137	306	443	4.1%	3.8%	3.8%	
Current smoker	293	994	1,287	10.3%	13.1%	12.4%	
Total	3,009	7,092	10,101	100.0%	100.0%	100.0%	
**Fast food consumption**							<0.001
None	1,999	2,263	4,262	65.8%	30.1%	39.1%	
1–2 times	631	2,845	3,476	26.5%	45.7%	40.9%	
More than twice	147	1,236	1,383	7.7%	24.1%	20.0%	
Total	2,777	6,344	9,121	100.0%	100.0%	100.0%	
**Daily intake of fruits**							<0.001
0–2 serves	2,769	6,600	9,369	94.2%	96.4%	95.9%	
3–4 serves	75	164	239	2.4%	2.4%	2.4%	
5 or more	90	105	195	3.3%	1.2%	1.7%	
Total	2,934	6,869	9,803	100.0%	100.0%	100.0%	
**Daily intake of vegetables**							<0.001
0–2 serves	2,391	6,120	8,511	86.5%	92.8%	91.3%	
3–4 serves	204	285	489	6.6%	3.8%	4.5%	
5 or more	141	201	342	6.9%	3.4%	4.3%	
Total	2,736	6,606	9,342	100.0%	100.0%	100.0%	
**Daily TV watching**							<0.001
0- less than 2 h	2,412	5,396	7,808	85.8%	81.0%	82.2%	
2- less than 4 h	284	888	1,172	9.4%	12.6%	11.8%	
4 h or more	105	459	564	4.8%	6.5%	6.1%	
Total	2,801	6,743	9,544	100.0%	100.0%	100.0%	
**Physical activity**							<0.001
Not active	1,623	3,737	5,360	71.6%	60.5%	63.2%	
Low	213	650	863	9.1%	12.3%	11.5%	
Moderate	249	784	1,033	11.4%	15.0%	14.1%	
High	165	622	787	7.9%	12.2%	11.1%	
Total	2,250	5,793	8,043	100.0%	100.0%	100.0%	
**Attitude toward fast food**							<0.001
Not healthy	2,486	5,961	8,447	84.7%	81.9%	82.6%	
Healthy	108	555	663	4.2%	9.8%	8.4%	
Do not know	417	583	1,000	11.1%	8.3%	9.0%	
Total	3,011	7,099	10,110	100.0%	100.0%	100.0%	
**Diabetes**							<0.001
No	2,375	6,584	8,959	85.6%	95.6%	93.2%	
Yes	500	396	896	14.4%	4.4%	6.8%	
Total	2,875	6,980	9,855	100.0%	100.0%	100.0%	

A linear trend was observed for age with each categorical decrease in consumption. The highest percentage (48.4%) of the SSB consumer belonged to the age group 15–24 years, whereas the proportion was the lowest (2.1%) for the age group 65 years and above. The consumers of SSB mostly resided in Riyadh (25.1%), followed by the Western Region (21.1%) and the Eastern Region (17.9%). Among the SSB consumers, about 58% of respondents had an education level of intermediate, secondary, or technical, while about 21% of the consumers had tertiary education. The highest proportion (20.5%) of consumers belonged to the income group of 3,000–5,000 Saudi Riyal (SR). SSB consumption was highly prevalent among participants who were not in the labor force (57.2%).

Table 3 shows the association of weekly SSB consumption with study variables in terms of AORs. The likelihood of consuming SSB per week was decreased as the respondent's age increased. For example, the odd of weekly SSB intake was about 80% lower among the oldest group (65 years and above) than the youngest groups (15–24 years). The likelihood of weekly SSB consumption was significantly higher among men (AOR: 1.42, 95% CI: 1.25–1.61). Compared with residents of the Riyadh region, respondents living in other regions (except Al Bahah) had significantly lower odds of SSB consumption per week. Among the socioeconomic variables, income and employment status were significantly associated with the probability of weekly consumption of SSB. The likelihood of weekly SSB consumption was lower (AOR: 0.77, 95% CI: 0.62–0.96) among individuals earning more than 15,000 SR compared to individuals earning an income of less than 3,000 SR.

**Table 3:** Association of weekly sugar-sweetened beverages (SSBs) consumption and study variables.

	**SSB consumption in a week**		
	**(any amount** **=** **1, none** **=** **0)**		
**Variables**	**AOR**	**95% CI**	***p* value**
**Age (Ref: 15–24 years)**			
25–34 years	0.72	(0.58–0.90)	<0.001
35–44 years	0.49	(0.39–0.62)	<0.001
45–54 years	0.35	(0.27–0.45)	<0.001
55–64 years	0.23	(0.17–0.30)	<0.001
65+ years	0.20	(0.15–0.27)	<0.001
**Male (Ref: Female)**	1.42	(1.25–1.61)	<0.001
**Marital status (Ref: Married)**			
Separated/Widowed/Divorced	0.91	(0.76–1.08)	0.27
Never married	1.49	(1.23–1.81)	<0.001
**Region (Ref: Riyadh)**			
Western region	0.88	(0.73–1.07)	0.20
Madinah	0.32	(0.26–0.40)	<0.001
Qaseem	0.51	(0.40–0.66)	<0.001
Eastern region	0.77	(0.62–0.96)	0.02
Asser/Bisha	0.70	(0.57–0.85)	<0.001
Tabouk	1.03	(0.79–1.35)	0.82
Hail	1.04	(0.83–1.31)	0.71
Northern borders	1.02	(0.79–1.32)	0.85
Jazan	0.55	(0.44–0.69)	<0.001
Najran	1.13	(0.89–1.42)	0.31
Albaha	1.25	(0.98–1.59)	0.07
AlJouf/Quriat	0.91	(0.70–1.19)	0.50
**Education (Ref: Primary)**			
Intermediate, secondary, or technical	1.14	(0.99–1.31)	0.07
Tertiary education	1.07	(0.90–1.28)	0.43
**Income (Ref:** ** <3,000 SR)**			
3,000 to less than 5,000 SR	1.05	(0.88–1.25)	0.60
5,000 to less than 7,000 SR	0.98	(0.81–1.18)	0.81
7,000 to less than 10,000 SR	0.73	(0.60–0.88)	<0.001
10,000 to less than 15,000 SR	0.81	(0.66–0.99)	0.04
≥15,000 SR	0.77	(0.62–0.96)	0.02
**Employment (Ref: Employed)**			
Unemployed	0.82	(0.68–0.98)	0.03
Out of labor force	0.88	(0.76–1.02)	0.08
**BMI (Ref:** ** <25)**			
BMI: 25–30	0.91	(0.80–1.03)	0.15
BMI: 30+	1.00	(0.87–1.14)	0.96
**Smoking status (Ref: never smoker)**			
Ex-smoker	1.10	(0.86–1.41)	0.45
Current smoker	1.12	(0.95–1.33)	0.18
**Fast food (Ref: No weekly consumption)**			
1–2 times in a week	2.82	(2.51–3.17)	<0.001
2+ times in a week	3.88	(3.18–4.73)	<0.001
**Fruit intake daily (Ref: 0–2 serves)**			
3–4 serves	1.05	(0.75–1.46)	0.78
5+ serves	0.71	(0.49–1.04)	0.08
**Vegetables intake daily (Ref: 0–2 serves)**			
3–4 serves	0.63	(0.51–0.78)	<0.001
5+ serves	0.63	(0.47–0.84)	<0.001
**Watching TV daily (Ref:** ** <2 h)**			
2–4 h	1.10	(0.94–1.29)	0.24
4+ h	1.31	(1.01–1.70)	0.04
**Physical activity (Ref: No)**			
Physical activity (low)	0.98	(0.82–1.17)	0.84
Physical activity (moderate)	0.89	(0.74–1.05)	0.17
Physical activity (high)	0.97	(0.79–1.19)	0.75
**Attitude about fast food (Ref: Not healthy)**			
Healthy	1.32	(1.02–1.69)	0.03
Do not know	0.87	(0.74–1.03)	0.10
**Diabetes (Ref: No)**	0.67	(0.57–0.79)	<0.001
**N**	10,118		

Table 3 also illustrates that the likelihood of SSB consumption in a typical week is about four times higher among participants who reported consuming fast food two times or more in a week. Participants who had more than three serves of vegetables daily were 37% less likely to consume SSB. Watching television for more than 4 h every day was associated with a higher likelihood of SSB consumption (AOR: 1.31, 95% CI: 1.01–1.70). Stratification of the sample between men and women and different age groups did not significantly change the results on the correlates of weekly SSB consumption (Supplementary Tables S1, S2).

Associations of daily SSB consumption and study factors among SSB consumers are presented in Table 4. Our results showed that the odds of daily SSB consumption were decreased with age. Men were more likely to consume SSB daily than women (AOR: 1.36, 95% CI: 1.18–1.56). Individuals living in different regions (except Al Bahah) of KSA had significantly lower odds of daily SSB consumption compared to people living in Riyadh. Respondents with intermediate, secondary, or technical education levels had a lower likelihood of consuming SSB daily than those with a primary level education (AOR: 0.83, 95% CI: 0.70–0.98). Individuals with an income of 10,000 to <15,000 SR had a higher likelihood of consuming SSB daily than individuals with an income of less than 3,000 SR (AOR: 1.25, 95% CI: 1.00–1.56). Respondents who were out of the labor force had higher odds of consuming SSB daily compared to employed respondents (AOR: 1.26 95% CI: 1.06–1.49).

**Table 4:** Association of daily vs. non-daily sugar-sweetened beverages (SSBs) consumption and study variables among SSB consumers.

	**SSB consumption**		
	**(daily** **=** **1, non-daily** **=** **0)**		
**Variables**	**AOR**	**95% CI**	***p* value**
**Age (Ref: 15–24 years)**			
25–34 years	0.72	(0.60–0.88)	<0.001
35–44 years	0.50	(0.40–0.63)	<0.001
45–54 years	0.31	(0.23–0.41)	<0.001
55–64 years	0.25	(0.17–0.36)	<0.001
65+ years	0.26	(0.17–0.39)	<0.001
**Male (Ref: Female)**	1.36	(1.18–1.56)	<0.001
**Marital status (Ref: Married)**			
Separated/Widowed/Divorced	0.92	(0.68–1.24)	0.58
Never married	1.37	(1.16–1.63)	<0.001
**Region (Ref: Riyadh)**			
Western region	1.37	(1.12–1.68)	<0.001
Madinah	1.95	(1.48–2.56)	<0.001
Qaseem	2.04	(1.51–2.76)	<0.001
Eastern Region	1.60	(1.26–2.04)	<0.001
Asser/Bisha	1.52	(1.19–1.96)	<0.001
Tabouk	1.16	(0.89–1.52)	0.28
Hail	1.31	(1.01–1.71)	0.04
Northern borders	3.02	(2.32–3.93)	<0.001
Jazan	2.27	(1.75–2.95)	<0.001
Najran	2.42	(1.89–3.10)	<0.001
Albaha	0.88	(0.66–1.17)	0.37
AlJouf/Quriat	1.39	(1.04–1.86)	0.03
**Education (Ref: Primary)**			
Intermediate, secondary, or technical	0.83	(0.70–0.98)	0.03
Tertiary education	0.92	(0.75–1.12)	0.39
**Income (Ref:** ** <3000 SR)**			
3,000 to less than 5,000 SR	1.01	(0.83–1.24)	0.93
5,000 to less than 7,000 SR	0.97	(0.79–1.20)	0.79
7,000 to less than 10,000 SR	1.07	(0.87–1.32)	0.51
10,000 to less than 15,000 SR	1.25	(1.00–1.56)	0.05
≥15,000 SR	0.96	(0.75–1.23)	0.73
**Employment (Ref: Employed)**			
Unemployed	1.17	(0.94–1.45)	0.16
Out of labor force	1.26	(1.06–1.49)	0.01
**BMI (Ref:** ** <25)**			
BMI: 25–30	0.93	(0.81–1.07)	0.32
BMI: 30+	1.16	(1.00–1.34)	0.05
**Smoking status (Ref: never smoker)**			
Ex-smoker	1.63	(1.25–2.14)	<0.001
Current smoker	1.90	(1.61–2.24)	<0.001
**Fast food (Ref: No weekly consumption)**			
1–2 times in a week	0.94	(0.82–1.09)	0.42
2+ times in a week	1.75	(1.48–2.08)	<0.001
**Fruit intake daily (Ref: 0–2 serves)**			
3–4 serves	1.28	(0.89–1.83)	0.18
5+ serves	0.95	(0.57–1.59)	0.85
**Vegetables intake daily (Ref: 0–2 serves)**			
3–4 serves	0.96	(0.72–1.27)	0.76
5+ serves	0.88	(0.62–1.26)	0.49
**Watching TV daily (Ref:** ** <2 h)**			
2–3 h	1.07	(0.90–1.27)	0.42
4+ h	1.65	(1.32–2.07)	<0.001
**Physical activity (Ref: No)**			
Physical activity (low)	1.05	(0.87–1.29)	0.60
Physical activity (moderate)	1.23	(1.03–1.46)	0.02
Physical activity (high)	1.73	(1.42–2.10)	<0.001
**Attitude about fast food (Ref: Not healthy)**			
Healthy	1.34	(1.09–1.64)	0.01
Do not know	1.03	(0.83–1.27)	0.80
**Diabetes (Ref: No)**	0.98	(0.74–1.31)	0.91
**N**	7,106		

We found that individuals with BMI over 30 were more likely to drink SSB daily compared to those with BMI less than 30 (AOR: 1.16, 95% CI: 1.00–1.34). Compared to individuals who never smoked, both previous and current smokers were more likely to consume SSB daily (AOR: 1.63, 95% CI: 1.25–2.14 and AOR: 1.90, 95% CI: 1.61–2.24). Individuals who consumed fast food more than two times a week were 75% more likely to report daily SSB consumption. Watching television for 4 h or more was significantly associated with a higher intake of SSB daily (AOR: 1.65, 95% CI: 1.32–2.07).

## Discussion

To the best of our knowledge, the present study is the first to highlight the determinants of SSB consumption in the KSA using data from nationally representative survey. The main strength of our study is to include a wide range of explanatory variables in the analysis. Our findings demonstrate that sedentary behaviors, such as frequently watching television and fast food consumption, were significantly associated with a higher likelihood of SSB intake. On the other hand, healthy eating behaviors decreased the likelihood of SSB consumption. We also found that younger groups were the most vulnerable among all age groups for SSB consumption. In addition, richer people had a lower likelihood of SSB consumption. However, there was no significant association between education and physical activity with a greater likelihood of SSB consumption.

Our findings demonstrated that demographic, socioeconomic, behavioral, and clinical factors were associated with SSB consumption. Approximately 71% of adults had consumed SSB in any given week, with 35.5% of adults consuming these drinks regularly, on a daily basis. Our findings followed the results from several earlier population-based studies in Saudi Arabia and Australia, showing that SSB consumption was higher among young adults, men, and low-income households (17, 37, 38). Although our study did not observe any association between SSB intake and adult educational attainment, existing evidence indicated that health literacy (i.e., individual capacity to comprehend basic health information required to make conscious health decisions) was linked to lower SSB intake (39). Women had significantly higher health literacy scores than men (21). Thus, low levels of health literacy could partially explain the positive relation between SSB intake among men in this study. This underscores the need to increase health literacy and provide clear and understandable health information among this population (17). The reason that lower-income individuals in this study were more likely to consume SSB was not clear and warranted further investigation.

Our findings indicate a linkage between behavioral factors and SSB consumption. In line with other studies, eating fast food was positively associated with SSB consumption (22, 38, 40, 41). Adults who had consumed fast food one time or two times a week had nearly three times the odds of being SSB consumers, and more frequent fast-food consumers (more than two times a week) had nearly four times the odds. This is particularly important given that several studies in Saudi Arabia indicated that Saudi children, adolescents, and adults are great consumers of fast food (42–45). The association between consuming fast food and takeaway meals and higher consumption of SSB have been well documented (17, 41). In this study, fast food was perceived as a “healthy food option” among this study population, implying that these adults might perceive SSB as somewhat “healthy.” This finding is critical given that adults with such perceptions are 30% more likely to be consumers of SSB in any given week or day. According to Miller et al. (17), Australian adults perceive SSB drinks as normal due to the availability, affordability, and promotion of these drinks. Taken together, strategies aimed at shaping adults' perception of fast food and SSB are needed. Evidence showed that on-pack health warning labels (46, 47) helped to promote an understanding of the potential risks of consuming SSB and decrease SSB sales (48).

Lack of knowledge as well about the content of SSB drinks might contribute to higher SSB intake (17). A recent study in Saudi Arabia showed that adults' awareness of the calorie content of SSB was related to lower SSB intake (49). Therefore, increasing awareness of the potential risks of fast food and SSB is imperative and could be the first step toward curbing both intakes (38). Although our analysis did not find a significant association between consumption of SSB and daily consumption of fruits, daily consumption of vegetables was positively associated. SSB consumption was higher among adults who consumed less than one serving per day of vegetables compared with those who consumed three or more serves per day. Our results showed that there was a specific eating behavior that correlated with SSB intake. A less healthy eating pattern is associated with an unhealthy beverage pattern (40). For instance, consumers of SSB are less likely to adhere to a prudent dietary pattern and are more likely to adhere to a fast food pattern (50).

In this study, we also observed that sedentary behavior, such as television viewing for long hours (more than 4 h a day), was associated with a greater likelihood of SSB consumption. Similar findings were found among both children and adolescents in other studies (14, 51, 52). The association between smoking and SSB intake among Saudi adults found in this study was in line with other studies (53). For example, a study in China demonstrated a positive association between smoking and higher consumption of SSB (54).

Despite the findings that SSB cluster with poor dietary behavior, smoking, and sedentary lifestyle, assessed clinical factors, such as BMI, were not related to SSB consumption in this study. This is in contrast with other studies, indicating that SSB intake could lead to weight gain (2). It has been proposed that being obese or overweight could make an individual more receptive to health promotion messages on dietary behaviors (30). This could also be valid among adults with diabetes. In the present study, people with diabetes were less likely to consume SSB compared to their non-diabetic counterparts. A USA study on the consumption of SSB among adults with type 2 diabetes found that 45% of adults with diabetes consumed SSB, consuming 202 calories and 47 g of sugar. However, adults with undiagnosed diabetes were significantly greater to have a higher SSB intake than adults with diabetes (1).

Further research is needed to assess changes in socioeconomic, behavioral, and clinical determinates of SSB intake among Saudi adults, following the implementation of the 50% excise tax on carbonated beverages in the country (55). Since the excise tax was promoted as an effective strategy for reducing the consumption of SSB, identifying determinants could be useful in the provision of customized population-based health interventions aimed at discouraging the consumption of SSB. Future studies should also monitor patterns of consumption among Saudi young, men, and lower-income individuals.

The analysis of this study has some limitations. The design of the study was cross-sectional. Therefore, it was not possible to draw causal conclusions from the observed significant relationships between outcome measures and their predictors. Another limitation was the use of self-reported consumption measure, which counted on the remembrance of participants without supplementary signaling to help recall. This problem might lead to an underestimated SSB consumption compared to an evaluation applying 24 h recall interview approach. Furthermore, this study could not compare the prevalence of SSB consumption between respondents and non-respondents. Due to the lack of data, this study could not consider energy intake while assessing the BMI and SSB consumption association.

## Conclusions

The results of nationally representative data demonstrated that socio-demographic, behavioral, and clinical factors are strong determinants of consumption of SSB among Saudi adults. The findings of this study have established essential population-based evidence to inform public health efforts to adopt effective strategies to reduce the consumption of SSB in the country. Interventions directed toward education on the adverse health effects of these drinks are needed to reduce SSB intake of adults.

## Data Availability Statement

The datasets generated and/or analyzed during the current study are not publicly available due to privacy, confidentiality and other restrictions. Access to data can be gained through the Ministry of Health in Saudi Arabia.

## Ethics Statement

This paper does not require ethical approval because we used a secondary data. Furthermore, the data is de-identified. The outcomes of the analysis does not allow re-identification and the use of data cannot result in any damage or distress.

## Author Contributions

All authors made substantial contributions to conception and design, acquisition of data, or analysis and interpretation of data, took part in drafting the article or revising it critically for important intellectual content, gave final approval of the version to be published, and agreed to be accountable for all aspects of the work.

## Funding

This research was funded by the Deanship of Scientific Research (DSR) at King Abdulaziz University, Grant Number G: 863-120-1441. The funders had no role in study design, data collection and analysis, decision to publish, or manuscript preparation.

## Conflict of Interest

The authors declare that the research was conducted in the absence of any commercial or financial relationships that could be construed as a potential conflict of interest.

## Publisher's Note

All claims expressed in this article are solely those of the authors and do not necessarily represent those of their affiliated organizations, or those of the publisher, the editors and the reviewers. Any product that may be evaluated in this article, or claim that may be made by its manufacturer, is not guaranteed or endorsed by the publisher.
