# Supplementary material for: Determinants of Sugar-Sweetened Beverage Consumption Among the Saudi Adults: Findings From a Nationally Representative Survey
Source: Front Nutr. 2022 Mar 22;9:744116. doi: 10.3389/fnut.2022.744116 (PMC8981208; doi:10.3389/fnut.2022.744116)
Supplement: Supplementary file 1 [file Table_1.DOCX]

**Supplementary Tables**

| **Table S1.** Logistic regression results on the correlates of sugar-sweetened beverage (SSB) consumption by male and female | | | | | | |
| --- | --- | --- | --- | --- | --- | --- |
|  | **Male** | | | **Female** | | |
| **Variables** | **AOR** | **95% CI** | **P value** | **AOR** | **95% CI** | **P value** |
| Age (Ref: 15-24 years) |  |  |  |  |  |  |
| 25-34 years | 0.64 | (0.44 - 0.94) | 0.02 | 0.79 | (0.61 - 1.03) | 0.08 |
| 35-44 years | 0.42 | (0.28 - 0.63) | <<0.0011 | 0.55 | (0.41 - 0.73) | <<0.0011 |
| 45-54 years | 0.28 | (0.19 - 0.43) | <<0.0011 | 0.41 | (0.30 - 0.57) | <<0.0011 |
| 55-64 years | 0.16 | (0.10 - 0.25) | <<0.0011 | 0.32 | (0.22 - 0.46) | <<0.0011 |
| 65+ years | 0.14 | (0.09 - 0.22) | <<0.0011 | 0.28 | (0.19 - 0.42) | <<0.0011 |
| Marital status (Ref: Married) |  |  |  |  |  |  |
| Separated/Widowed/Divorced | 1.17 | (0.79 - 1.74) | 0.43 | 0.79 | (0.64 - 0.98) | 0.03 |
| Never married | 1.35 | (0.99 - 1.84) | 0.06 | 1.51 | (1.18 - 1.94) | <<0.0011 |
| Region (Ref: Riyadh) |  |  |  |  |  |  |
| Western Region | 0.95 | (0.72 - 1.26) | 0.74 | 0.84 | (0.65 - 1.08) | 0.17 |
| Madinah | 0.37 | (0.27 - 0.50) | <0.001 | 0.29 | (0.21 - 0.39) | <0.001 |
| Qaseem | 0.56 | (0.38 - 0.82) | <0.001 | 0.48 | (0.34 - 0.67) | <0.001 |
| Eastern Region | 0.90 | (0.65 - 1.26) | 0.55 | 0.63 | (0.47 - 0.86) | <0.001 |
| Asser/Bisha | 0.75 | (0.55 - 1.01) | 0.06 | 0.66 | (0.51 - 0.87) | <0.001 |
| Tabouk | 0.87 | (0.60 - 1.26) | 0.45 | 1.23 | (0.86 - 1.75) | 0.25 |
| Hail | 1.12 | (0.80 - 1.58) | 0.50 | 1.04 | (0.75 - 1.44) | 0.81 |
| Northern Borders | 0.86 | (0.59 - 1.25) | 0.43 | 1.23 | (0.86 - 1.76) | 0.26 |
| Jazan | 0.53 | (0.37 - 0.75) | <0.001 | 0.56 | (0.41 - 0.75) | <0.001 |
| Najran | 1.09 | (0.77 - 1.55) | 0.62 | 1.17 | (0.85 - 1.60) | 0.34 |
| Albaha | 1.69 | (1.14 - 2.52) | 0.01 | 1.05 | (0.76 - 1.44) | 0.78 |
| AlJouf/Quriat | 1.03 | (0.68 - 1.57) | 0.89 | 0.83 | (0.59 - 1.17) | 0.29 |
| Education (Ref: Primary) |  |  |  |  |  |  |
| Intermediate, secondary, or technical | 1.02 | (0.82 - 1.27) | 0.86 | 1.28 | (1.06 - 1.55) | 0.01 |
| Tertiary education | 0.98 | (0.75 - 1.28) | 0.86 | 1.17 | (0.92 - 1.49) | 0.21 |
| Income (Ref: <3000 SR) |  |  |  |  |  |  |
| 3000 to less than 5000 SR | 0.83 | (0.62 - 1.10) | 0.20 | 1.18 | (0.93 - 1.51) | 0.18 |
| 5000 to less than 7000 SR | 0.81 | (0.60 - 1.10) | 0.17 | 1.06 | (0.82 - 1.37) | 0.68 |
| 7000 to less than 10000 SR | 0.63 | (0.47 - 0.86) | <0.001 | 0.76 | (0.58 - 0.99) | 0.04 |
| 10000 to less than 15000 SR | 0.65 | (0.47 - 0.90) | 0.01 | 0.91 | (0.69 - 1.20) | 0.50 |
| ≥15000 SR | 0.66 | (0.47 - 0.92) | 0.01 | 0.81 | (0.60 - 1.10) | 0.17 |
| Employment (Ref: Employed) |  |  |  |  |  |  |
| Unemployed | 0.64 | (0.47 - 0.88) | 0.01 | 0.85 | (0.66 - 1.08) | 0.18 |
| Out of labor force | 0.94 | (0.75 - 1.18) | 0.61 | 0.87 | (0.70 - 1.07) | 0.19 |
| BMI (Ref:< 25) |  |  |  |  |  |  |
| BMI: 25-30 | 0.89 | (0.74 - 1.07) | 0.22 | 0.91 | (0.76 - 1.10) | 0.34 |
| BMI: 30+ | 0.94 | (0.77 - 1.14) | 0.53 | 1.01 | (0.84 - 1.21) | 0.92 |
| Smoking status (Ref: never smoker) |  |  |  |  |  |  |
| Ex-smoker | 1.17 | (0.90 - 1.52) | 0.23 | 0.61 | (0.25 - 1.49) | 0.28 |
| Current smoker | 1.08 | (0.90 - 1.29) | 0.42 | 2.10 | (1.14 - 3.84) | 0.02 |
| Fast food (Ref: No weekly consumption) |  |  |  |  |  |  |
| 1-2 times in a week | 2.96 | (2.48 - 3.55) | <0.001 | 2.66 | (2.27 - 3.13) | <0.001 |
| 2+ times in a week | 4.01 | (3.02 - 5.31) | <0.001 | 3.66 | (2.77 - 4.84) | <0.001 |
| Fruits intake daily (Ref: 0-2 serves) |  |  |  |  |  |  |
| 3-4 serves | 1.16 | (0.76 - 1.78) | 0.49 | 0.94 | (0.58 - 1.53) | 0.80 |
| 5+ serves | 0.82 | (0.48 - 1.40) | 0.47 | 0.57 | (0.34 - 0.97) | 0.04 |
| Vegetables intake daily (Ref: 0-2 serves) |  |  |  |  |  |  |
| 3-4 serves | 0.55 | (0.41 - 0.74) | <0.001 | 0.82 | (0.59 - 1.14) | 0.24 |
| 5+ serves | 0.57 | (0.37 - 0.88) | 0.01 | 0.74 | (0.50 - 1.08) | 0.12 |
| Watching tv daily (Ref: < 2 hours) |  |  |  |  |  |  |
| 2- 3 hours | 1.09 | (0.86 - 1.39) | 0.47 | 1.11 | (0.90 - 1.38) | 0.34 |
| 4+ hours | 1.32 | (0.94 - 1.87) | 0.11 | 1.33 | (0.92 - 1.92) | 0.14 |
| Physical activity (Ref: No) |  |  |  |  |  |  |
| Physical activity (low) | 0.97 | (0.76 - 1.24) | 0.80 | 0.96 | (0.73 - 1.26) | 0.75 |
| Physical activity (moderate) | 0.80 | (0.64 - 1.00) | 0.05 | 0.99 | (0.75 - 1.31) | 0.95 |
| Physical activity (high) | 0.88 | (0.68 - 1.15) | 0.36 | 1.05 | (0.74 - 1.48) | 0.79 |
| Attitude about fast food (Ref: Not healthy) |  |  |  |  |  |  |
| Healthy | 1.68 | (1.14 - 2.47) | 0.01 | 1.14 | (0.83 - 1.55) | 0.42 |
| Do not know | 0.94 | (0.72 - 1.22) | 0.64 | 0.83 | (0.67 - 1.03) | 0.09 |
| Diabetes (Ref: No) |  |  |  |  |  |  |
| Yes | 0.63 | (0.50 - 0.79) | <0.001 | 0.74 | (0.57 - 0.95) | 0.02 |
| N | 5,028 | | | 5,090 | | |

^Notes: AOR is adjusted odds ratio and 95 % confidence interval in parentheses.^

| **Table S2.** Logistic regression results on the correlates of sugar-sweetened beverage (SSB) consumption (15-24 years age group vs 25 years+ age group) | | | | | | |
| --- | --- | --- | --- | --- | --- | --- |
|  | **15-24 years** | | | **25+ years** | | |
| **Variables** | **AOR** | **95% CI** | **P value** | **AOR** | **95% CI** | **P value** |
| Sex (Ref: Female) |  |  |  |  |  |  |
| Male | 1.67 | (1.19 - 2.34) | <0.001 | 1.02 | (0.89 - 1.17) | 0.76 |
| Marital status (Ref: Married) |  |  |  |  |  |  |
| Separated/Widowed/Divorced | 0.36 | (0.10 - 1.28) | 0.11 | 0.71 | (0.59 - 0.84) | <0.001 |
| Never married | 1.24 | (0.84 - 1.82) | 0.28 | 1.80 | (1.45 - 2.23) | <0.001 |
| Region (Ref: Riyadh) |  |  |  |  |  |  |
| Western Region | 0.53 | (0.30 - 0.93) | 0.03 | 0.87 | (0.72 - 1.06) | 0.16 |
| Madinah | 0.24 | (0.13 - 0.45) | <0.001 | 0.33 | (0.26 - 0.42) | <0.001 |
| Qaseem | 0.57 | (0.29 - 1.10) | 0.09 | 0.47 | (0.36 - 0.62) | <0.001 |
| Eastern Region | 0.61 | (0.32 - 1.16) | 0.13 | 0.81 | (0.64 - 1.02) | 0.08 |
| Asser/Bisha | 0.57 | (0.32 - 1.04) | 0.07 | 0.73 | (0.60 - 0.90) | <0.001 |
| Tabouk | 0.79 | (0.34 - 1.83) | 0.58 | 1.08 | (0.83 - 1.40) | 0.57 |
| Hail | 1.17 | (0.52 - 2.64) | 0.70 | 1.03 | (0.80 - 1.31) | 0.84 |
| Northern Borders | 1.47 | (0.60 - 3.56) | 0.40 | 0.96 | (0.73 - 1.25) | 0.75 |
| Jazan | 0.27 | (0.15 - 0.46) | <0.001 | 0.66 | (0.52 - 0.85) | <0.001 |
| Najran | 1.00 | (0.49 - 2.05) | 1.00 | 1.21 | (0.95 - 1.55) | 0.12 |
| Albaha | 1.56 | (0.64 - 3.77) | 0.33 | 1.13 | (0.88 - 1.46) | 0.34 |
| AlJouf/Quriat | 0.65 | (0.32 - 1.34) | 0.25 | 0.97 | (0.73 - 1.29) | 0.84 |
| Education (Ref: Primary) |  |  |  |  |  |  |
| Intermediate, secondary, or technical | 1.56 | (1.07 - 2.28) | 0.02 | 1.50 | (1.30 - 1.72) | <0.001 |
| Tertiary education | 1.34 | (0.77 - 2.31) | 0.30 | 1.47 | (1.23 - 1.74) | <0.001 |
| Income (Ref: <3000 SR) |  |  |  |  |  |  |
| 3000 to less than 5000 SR | 1.08 | (0.64 - 1.82) | 0.77 | 1.11 | (0.92 - 1.34) | 0.26 |
| 5000 to less than 7000 SR | 0.73 | (0.43 - 1.24) | 0.24 | 1.09 | (0.89 - 1.34) | 0.40 |
| 7000 to less than 10000 SR | 0.43 | (0.26 - 0.71) | <0.001 | 0.87 | (0.70 - 1.07) | 0.18 |
| 10000 to less than 15000 SR | 0.59 | (0.35 - 1.02) | 0.06 | 0.83 | (0.67 - 1.03) | 0.10 |
| ≥15000 SR | 0.61 | (0.33 - 1.15) | 0.13 | 0.75 | (0.60 - 0.94) | 0.01 |
| Employment (Ref: Employed) |  |  |  |  |  |  |
| Unemployed | 0.52 | (0.28 - 1.00) | 0.05 | 0.74 | (0.62 - 0.89) | <0.001 |
| Out of labor force | 1.14 | (0.64 - 2.04) | 0.66 | 0.68 | (0.59 - 0.79) | <0.001 |
| BMI (Ref:< 25) |  |  |  |  |  |  |
| BMI: 25-30 | 1.23 | (0.86 - 1.77) | 0.26 | 0.84 | (0.73 - 0.97) | 0.01 |
| BMI: 30+ | 0.98 | (0.65 - 1.48) | 0.93 | 0.93 | (0.81 - 1.07) | 0.29 |
| Smoking status (Ref: never smoker) |  |  |  |  |  |  |
| Ex-smoker | 0.66 | (0.24 - 1.84) | 0.43 | 1.09 | (0.85 - 1.39) | 0.50 |
| Current smoker | 1.25 | (0.66 - 2.39) | 0.50 | 1.20 | (1.01 - 1.43) | 0.04 |
| Fast food (Ref: No weekly consumption) |  |  |  |  |  |  |
| 1-2 times in a week | 2.60 | (1.87 - 3.61) | <0.001 | 3.02 | (2.66 - 3.42) | <0.001 |
| 2+ times in a week | 3.88 | (2.48 - 6.08) | <0.001 | 4.36 | (3.50 - 5.44) | <0.001 |
| Fruits intake daily (Ref: 0-2 serves) |  |  |  |  |  |  |
| 3-4 serves | 1.36 | (0.38 - 4.92) | 0.64 | 1.01 | (0.73 - 1.41) | 0.94 |
| 5+ serves | 0.40 | (0.11 - 1.42) | 0.16 | 0.73 | (0.50 - 1.08) | 0.11 |
| Vegetables intake daily (Ref: 0-2 serves) |  |  |  |  |  |  |
| 3-4 serves | 0.94 | (0.45 - 1.99) | 0.87 | 0.60 | (0.48 - 0.75) | <0.001 |
| 5+ serves | 1.06 | (0.42 - 2.66) | 0.90 | 0.60 | (0.44 - 0.81) | <0.001 |
| Watching TV daily (Ref: < 2 hours) |  |  |  |  |  |  |
| 2- 3 hours | 0.86 | (0.57 - 1.28) | 0.44 | 1.20 | (1.00 - 1.42) | 0.04 |
| 4+ hours | 1.02 | (0.57 - 1.83) | 0.94 | 1.45 | (1.10 - 1.90) | 0.01 |
| Physical activity (Ref: No) |  |  |  |  |  |  |
| Physical activity (low) | 1.26 | (0.76 - 2.11) | 0.37 | 0.94 | (0.78 - 1.14) | 0.52 |
| Physical activity (moderate) | 1.35 | (0.85 - 2.15) | 0.21 | 0.85 | (0.70 - 1.02) | 0.09 |
| Physical activity (high) | 1.56 | (0.87 - 2.81) | 0.13 | 0.96 | (0.76 - 1.20) | 0.70 |
| Attitude about fast food (Ref: Not healthy) |  |  |  |  |  |  |
| Healthy | 2.04 | (1.03 - 4.02) | 0.04 | 1.25 | (0.97 - 1.61) | 0.09 |
| Do not know | 0.73 | (0.46 - 1.15) | 0.18 | 0.85 | (0.72 - 1.02) | 0.08 |
| Diabetes (Ref: No) |  |  |  |  |  |  |
| Yes | 0.41 | (0.10 - 1.65) | 0.21 | 0.54 | (0.46 - 0.63) | <0.001 |
| N | 2,278 | |  | 7,824 | |  |
| ^Notes: AOR is adjusted odds ratio and 95 % confidence interval in parentheses.^ | | | | | | |
